# The immediate effects of local and adjacent acupuncture on the tibialis anterior muscle: a human study

**DOI:** 10.1186/1749-8546-3-17

**Published:** 2008-12-18

**Authors:** Larissa Araujo Costa, João Eduardo de Araujo

**Affiliations:** 1 Acupuncture specialization course, Instituto Paulista de Estudos Sistêmicos (IPES), Praça Boaventura Ferreira da Rosa 384, Ribeirão Preto (SP) 14049-900, Brazil; 2 Laboratory of Bioengineering, Neuropsychobiology and Motor Behavior, Department of Biomechanics, Medicine and Rehabilitation of the Locomotor System, School of Medicine – University of São Paulo, Ribeirão Preto (USP-RP), Avenida dos Bandeirantes 3900, Ribeirão Preto (SP) 14049-900, Brazil

## Abstract

**Background:**

This study compares the immediate effects of local and adjacent acupuncture on the tibialis anterior muscle and the amount of force generated or strength in Kilogram Force (KGF) evaluated by a surface electromyography.

**Methods:**

The study consisted of a single blinded trial of 30 subjects assigned to two groups: local acupoint (ST36) and adjacent acupoint (SP9). Bipolar surface electrodes were placed on the tibialis anterior muscle, while a force transducer was attached to the foot of the subject and to the floor. An electromyograph (EMG) connected to a computer registered the KGF and root mean square (RMS) before and after acupuncture at maximum isometric contraction. The RMS values and surface electrodes were analyzed with Student's t-test.

**Results:**

Thirty subjects were selected from a total of 56 volunteers according to specific inclusion and exclusion criteria and were assigned to one of the two groups for acupuncture. A significant decrease in the RMS values was observed in both ST36 (t = -3.80, *P *= 0,001) and SP9 (t = 6.24, *P *= 0.001) groups after acupuncture. There was a decrease in force in the ST36 group after acupuncture (t = -2.98, *P *= 0.006). The RMS values did not have a significant difference (t = 0.36, *P *= 0.71); however, there was a significant decrease in strength after acupuncture in the ST36 group compared to the SP9 group (t = 2.51, *P *= 0.01). No adverse events were found.

**Conclusion:**

Acupuncture at the local acupoint ST36 or adjacent acupoints SP9 reduced the tibialis anterior electromyography muscle activity. However, acupuncture at SP9 did not decrease muscle strength while acupuncture at ST36 did.

## Background

Acupuncture is an integral part of Chinese medicine and is widely practiced in China and other countries [1] to treat conditions [2] and diseases such as myofascial pain syndrome [3], muscle spasticity after stroke [4], knee osteoarthritis [5,6] and lower back pain [7].

According to Chinese medicine, there is a network of meridians (*jingluo*) connecting functional organs in the human body. Acupuncture at specific acupoints along the meridians exerts therapeutic effects on nearby and/or distant regions. Previous studies [1-4,8-10] reported physiological effects of acupuncture.

Recent neural-imaging, pharmacological and electromyography data showed that some of the acupuncture effects were likely to be mediated by the activation of areas within the central nervous system (CNS) [8]. It was suggested that the hypothalamus-pituitary-adrenal axis and its neurotransmitters are associated with the excitability of the CNS observed under acupuncture [8]. Neural-imaging techniques also demonstrated some long-term plastic changes in the CNS after somatic sensory stimulation of the afferent fibers by acupuncture [1]. Furthermore, endocrine and immunologic responses of athletes to acupuncture were related to the stimulation of the sympathetic nervous system [9]. Bilateral motor unit activation was observed during unilateral acupuncture of active myofascial trigger points (MTrPs) via the CNS [3] and it was speculated that some acupoints as MTrPs caused the local increase of end plate noise (EPN) during acupuncture [10]. A decrease in electromyography activity has been reported in masticator muscles and spastic wrist flexor muscles of stroke survivors after acupuncture [2,4]. Another study on anatomical localization of acupoints identified muscle spindles and other mechanoreceptors at the sites [11] known to influence excitability in human studies. Since ST and SP are joined meridians that commonly control muscle energy, it would be interesting to investigate how stimulations at different points on these two meridians could differentially affect the mechanical and electrical properties of a muscle.

This study aims to compare the immediate physiological effects of acupuncture on the local ST36 (*Zusanli*) and adjacent SP9 (*Yinlingquan*) acupoints in the tibialis anterior muscle so that we can verify whether acupuncture can modulate the electric stimulation and strength in the local and adjacent (relatively distant) regions of this muscle.

## Methods

### Subjects

Twelve male and 18 female subjects aged 18–25 years were recruited from the University of Sao Paulo between August and October in 2007. All subjects were healthy. The exclusion criteria were lower limb pain, trauma history, neuromuscular problems, pregnancy or any type of panic reaction to needles.

**Figure 1:**
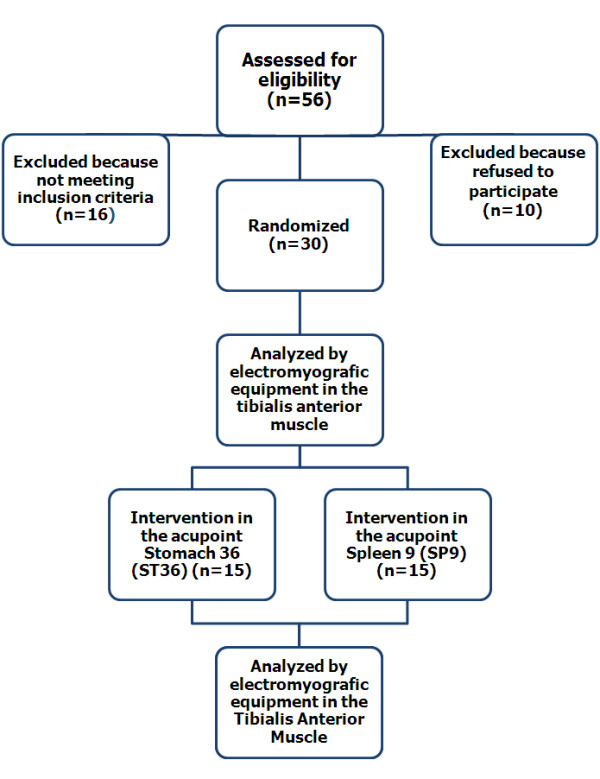
**Flow diagram of the local and adjacent acupuncture**. The diagram also includes the number of volunteers who were excluded from the trial.

The subjects were assigned with the help of an independent researcher who did not know the aim of this study. The assignment was mainly based on the subject's own choice to join either the ST36 group or the SP9 group until both groups had 15 subjects (Figure 1).

The trial was carried out in the Laboratory of Bioengineering, Neuropsychobiology and Motor Behavior at the University of Sao Paulo between August and November in 2007. The Committee of Ethics in Research at the University of Sao Paulo approved the study methods and procedures.

### Treatment

Acupuncture was performed by an acupuncturist with a certificate by the Federal Physical Therapy Council and Brazilian Society of Physical Therapists and Acupuncturists. Sterile and disposable acupuncture needles with tubes (0.25 mm in diameter, 40 mm in length, DongBang, Korea) were used. The local ST36 and adjacent (relatively distant) SP9 were used because ST36 is on the tibialis anterior muscle and SP9 was reported to be also effective in treating the muscular system [12]. The 'snapping technique' (without needle rotation) was employed at an insertion depth of approximately 1.5 cm. The duration of a treatment session was 20 minutes. The needles were stimulated during the first two minutes and for one minute at the fifth, tenth, fifteenth, nineteenth minutes of treatment. Each patient received only one treatment session.

### Evaluation

Both groups were evaluated with surface electromyography of the tibialis anterior muscle before and after the acupuncture session.

### Instrument

A six-channel surface electromyography machine with a force transducer (400c-200c model, EMG System of Brazil Co, Brazil) connecting to a Toshiba laptop computer was used. Disposable double superficial silver-silver chloride pre-gelled snap electrodes (10 mm in diameter and inter-electrode distance, EMG System of Brazil Co, Brazil) were employed in the study.

### Procedure

Before the electrodes were placed, skin was shaved and cleaned with 70% alcohol. Electrodes were placed while the subject sat on a treatment table according to the anatomical references and procedures in previous studies [13,14]. The tibialis anterior belly was located by palpation during active dorsiflexion. The electrode site was two centimeters distal and lateral from the tibial tubercle. An electrode of reference (ground) was placed on the subject's radial styloid process; on the same side the tibialis anterior electrode was placed. These electrodes remained in place until the acupuncture treatment was done.

A force transducer was attached to the treatment table support and the dominant-side foot by a non-elastic material. The foot remained in slight plantar flexion due to the limitation in dorsiflexion range of motion (ROM) that the non-elastic material induced. It was necessary to have a decreased dorsiflexion ROM, to allow the isometric contraction of the tibialis anterior muscle. The subjects were asked to report any discomfort and ask questions during the procedure.

The electromyography and force transducer data of the tibialis anterior muscle were collected during the rest position and the isometric dorsiflexion was performed before and after acupuncture. The subjects were instructed to apply the maximum possible strength during the dorsiflexion and avoid any movement in the knees or hips.

Both ST36 and SP9 groups were submitted to the same procedures: (1) electromyography registration of rest position, (2) electromyography registration of the isometric dorsiflexion, (3) acupuncture at either ST36 or SP9 acupoints for 20 minutes, (4) electromyography registration of isometric dorsiflexion.

To ensure the quality of the signal, we determined the root mean square (RMS) of the rest position at 10% to 15% of the isometric contraction according to a previous study [15]. The RMS values were obtained from three contractions accomplished by the subjects. If the rest RMS was higher than the selected value, the subject was excluded from the study. Electromyography and force transducer data were compared before and after acupuncture during isometric dorsiflexion of the dominant-side foot.

### Statistical analysis

RMS and KGF values obtained for each subject before and after acupuncture. The ratios of after-acupuncture values to before-acupuncture values were presented in percentage. The distribution of percentage data was confirmed by Kolmogorov-Smirnov test to have no significant deviation from a normal distribution. All data were analyzed by two independent researchers in our laboratory who were blinded to the group assignment. The data were reported as mean ± standard deviation (SD). Comparison between before-acupuncture and after-acupuncture conditions of same subjects was conducted by paired t-test. The differences between the groups after acupuncture were analyzed by non-paired t-test. All statistical analyses were conducted with SigmaStat 3.1 software. The results of all tests (including Kolmogorov-Smirnov, paired t-test, and non-paired t-test) with *P *< 0.05 were considered to be statistically significant.

## Results

Thirty subjects selected from a total of 56 volunteers were assigned to one of the two groups for acupuncture. The remaining 26 volunteers were excluded according to the exclusion criteria.

**Figure 2:**
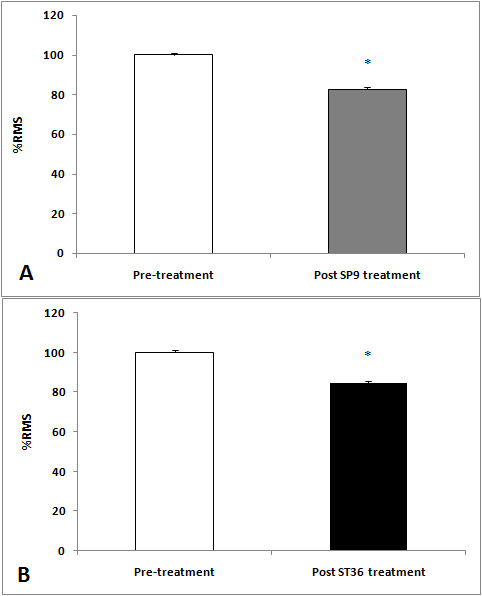
**Electromyography activities in root mean square (RMS) percentage for the SP9 (A) and ST36 (B) groups before and after acupuncture**. Data were reported as mean ± SD. *Statistically significant differences, in comparison to the pre- and post-SP9 or ST36 treatment values, according to paired t-test (*P *= 0.001) in A and (*P *= 0.002) in B. N = 15 for each group, Pre: local and adjacent point groups before acupuncture, Post ST36 treatment: local point group after acupuncture, Post SP9 treatment: adjacent point group after acupuncture.

**Figure 3:**
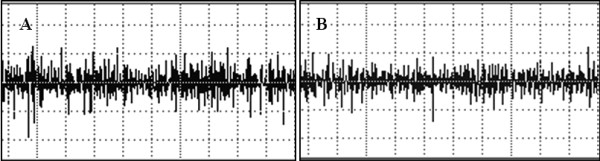
**Electromyography (EMG) of a single subject in the local point ST36 group**. A = EMG acupuncture. B = EMG after acupuncture. In the x axis the duration is 5 seconds. In the y axis the amplitude scale is 308 μV in A and 102 μV in B.

**Figure 4:**
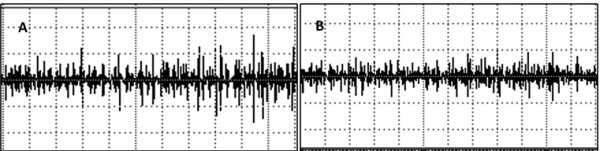
**Electromyography (EMG) of a single subject in the adjacent point SP9 group**. A = EMG before acupuncture. B = EMG after acupuncture. In the x axis the duration is 5 seconds. In the y axis the amplitude scale is 33 μV in A and 25 μV in B.

**Figure 5:**
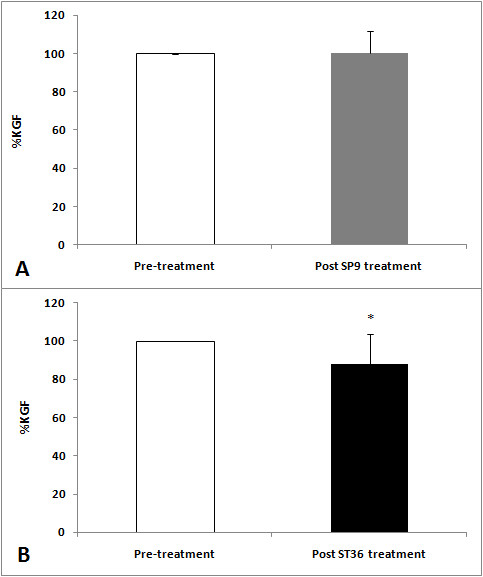
**Muscle strength (KGF) results of the SP9 (A) and ST36 (B) groups before and after acupuncture**. Data are reported as mean ± SD. * The values between the two groups after acupuncture are statistically different (*P *= 0.01). N = 15 for each group, Pre: local and adjacent point groups before acupuncture, Post ST36 treatment: local point group after acupuncture, Post SP9 treatment: adjacent point group after acupuncture.

**Figure 6:**
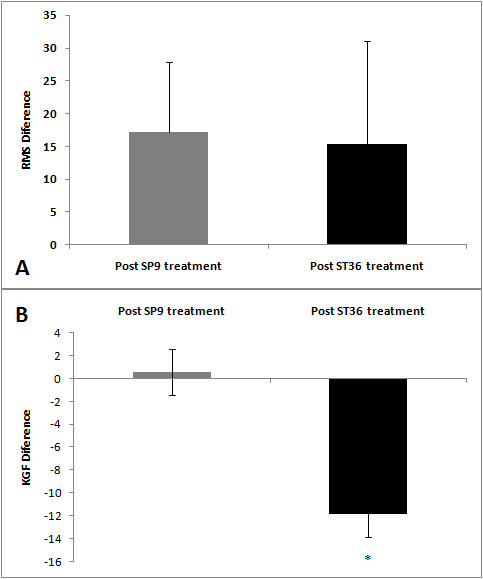
**Electromyography activities in root mean square (RMS) percentage (A) and muscle strength (KGF) (B) in the SP9 and ST36 groups before acupuncture**. Data are reported as mean ± SD. * The values between the two groups after acupuncture are statistically different (*P *= 0.01). N = 15 for each group, Pre: local and adjacent point groups before acupuncture, Post ST36 treatment: local point group after acupuncture, Post SP9 treatment: adjacent point group after acupuncture.

A significant decrease in the RMS values was observed in both ST36 (t = -3.80, *P *= 0,001) and SP9 (t = 6.24, *P *= 0.001) groups after acupuncture (Figures 2, 3 and 4). There was a decrease in force in the ST36 group after acupuncture (t = -2.98, *P *= 0.006) (Figure 5). The RMS values did not have a significant difference (t = 0.36, *P *= 0.71) (Figure 6); however, there was a significant decrease in strength after acupuncture in the ST36 group compared to the SP9 group (t = 2.51, *P *= 0.01). No adverse events were found.

## Discussion

The present survey found differences among the electromyography activities of the tibialis anterior muscle before and after acupuncture at acupoints ST36 and SP9 at the isometric contraction. The decrease in RMS values after acupuncture indicates that the electrical activity generated by the tibialis anterior muscle was reduced to allow relaxation [13,14].

RMS values in both ST36 and SP9 groups decreased after acupuncture. There were no significant differences in the RMS values between the two groups after acupuncture. These findings support that acupuncture can influence muscle activity and strength.

Few studies were reported on acupuncture on muscle strength [16,17]. In the present study, the local point group (ST36) showed a significant decrease in post acupuncture muscle strength value (KGF) while the adjacent point group (SP9) did not change in muscle strength. This new finding suggests that acupuncture at the local acupoint ST36 may influence the reflex loop of the tibialis anterior muscle thereby decreasing muscle strength. The finding that acupuncture at the adjacent point SP9 did not decrease muscle strength may indicate that SP9 did not act on the same reflex loop as ST36 did.

Further investigations are required to answer questions such as whether the stimulated muscle activities by acupuncture is sustainable after treatment and whether the acupuncture response in the tibialis anterior muscle may also occur in other muscles.

## Conclusion

Acupuncture at the local acupoint ST36 or adjacent acupoints SP9 reduced the tibialis anterior electromyography muscle activity. However, acupuncture at SP9 did not decrease muscle strength while acupuncture at ST36 did.

## Abbreviations

CNS: central nervous system; EPN: end plate noise; EMG: electromyography; KGF: Kilogram Force; MTrPs: myofascial trigger points; ROM: range of motion; RMS: root mean square; SD: standard deviation; SP9: spleen 9 (*Yinlingquan*); ST36: stomach 36 (*Zusanli*)

## Competing interests

The authors declare that they have no competing interests.

## Authors' contributions

LAC helped the study design and conducted the trial. JEA conceived and coordinated the study, conducted the trial, statistical analysis and wrote the manuscript. Both authors read and approved the final version of the manuscript.

## References

[B1] Maioli C, Falciati L, Marangon M, Perini S, Losio A (2006). Short- and long-term modulation of upper limb motor evoked potentials induced by acupuncture. Eur J Neuros.

[B2] Sousa RA, Semprini M, Vitti M, Borsato MC, Hallack Regalo SC (2007). Electromyographic evaluation of the masseter and temporal muscles activity in volunteers submitted to acupuncture. Electromyogr Clin Neurophysiol.

[B3] Audette JF, Wang F, Smith H (2004). Bilateral activation of motor unit potentials with unilateral needle stimulation of active myofascial trigger points. Am J Phys Med Rehabil.

[B4] Mukherjee M, McPeak LK, Redford JB, Sun C, Liu W (2007). The effect of electro-acupuncture on spasticity of the wrist joint in chronic stroke survivors. Arch Phys Med Rehabil.

[B5] Berman BM, Lao L, Langenberg P, Lee WL, Gilpin AMK, Hochberg MC (2004). Effectiveness of acupuncture as adjunctive therapy in osteoarthritis of the knee. Ann Intern Med.

[B6] Sangdee C, Teekachunhatean S, Sananpanich K, Sugandhavesa N, Chiewchantanakit S, Pojchamarnwiputh S, Jayasvasti S (2002). Electroacupuncture versus Diclofenac in symptomatic treatment of osteoarthritis of the knee: a randomized controlled trial. BMC Compl Altern Med.

[B7] Vas J, Perea-Milla E, Mendez C, Silva LC, Galante AH, Regules JMA, Barquin DMM, Aguilar I, Faus V (2006). Efficacy and safety of acupuncture for the treatment of non-specific acute low back pain: a randomised controlled multicentre trial protocol. BMC Compl Altern Med.

[B8] Cho ZH, Hwang SC, Wong EK, Son YD, Kang CK, Park TS, Bai SJ, Kim YB, Lee YB, Sung KK, Lee BH, Shepp LA, Min KT (2006). Neural substrates, experimental evidences and functional hypothesis of acupuncture mechanisms. Acta Neurol Scand.

[B9] Akimoto T, Nakahori C, Aizawa K, Kimura F, Fukubayashi T, Kono I (2003). Acupuncture and responses of immunologic and endocrine markers during competition. Med Sci Sports Exerc.

[B10] Kao MJ, Hsieh YL, Kuo FJ, Hong CZ (2006). Electrophysiological assessment of acupuncture points. Am J Phys Med Rehabil.

[B11] Lo YL, Cui SL, Fook-Chong S (2005). The effect of acupuncture on motor cortex excitability and plasticity. Neurosc Letters.

[B12] Wang K, Liu J (1989). Needling sensation receptor of an acupoint supplied by the median nerve – studies of their electrophysiological characteristics. Am J Chin Med.

[B13] Wen TS (1995). Classic Chinese Acupuncture.

[B14] Turker KS (1993). Electromyography: some methodological problems and issues. Phys Ther.

[B15] De Luca CJ (1997). The use of surface electromyography in biomechanics. J Appl Biomech.

[B16] Toma K, Conatser RR, Gilders RM, Hagerman FC (1998). The effects of acupuncture needle stimulation on skeletal muscle activity and performance. J Strength Cond Res.

[B17] Pelham TW, Holt LE, Stalker R (2001). Acupuncture in human performance. J Strength Cond Res.

